# Parapharyngeal space metastasis from squamous cell carcinoma: indications and limits of different surgical approaches

**DOI:** 10.1097/MOO.0000000000001029

**Published:** 2024-12-19

**Authors:** Matteo Fermi, Carlotta Liberale, Gabriele Molteni

**Affiliations:** a Department of Otorhinolaryngology - Head and Neck Surgery, IRCCS Azienda Ospedaliero-Universitaria di Bologna; b Department of Medical and Surgical Sciences (DIMEC), Alma Mater Studiorum - Università di Bologna, Bologna; c Unit of Otorhinolaryngology, Head & Neck Department, University of Verona, Verona, Italy

**Keywords:** parapharyngeal space, parapharyngeal space surgery, squamous cell carcinoma metastasis, transcervical–transparotid approach

## Abstract

**Purpose of review:**

The aim of this review is to investigate the most suitable surgical approach to managing parapharyngeal space (PPS) squamous cell carcinoma (SCC) metastasis.

**Recent findings:**

SCC metastasis in PPS are extremely rare. The PPS itself is a complex anatomical area, requiring extensive surgical experience and various surgical approaches for effective management. Several authors have attempted to systematize the surgical approaches to the PPS based on the anatomical location and histological nature of the lesions. However, there are currently few studies in the literature on the specific management of SCC metastases in the PPS, as these lesions are extremely rare.

**Summary:**

The treatment of SCC metastases in the PPS must be determined based on the individual patient. If the patient is a candidate for surgery, the surgical approach should be chosen based on the location of the metastases and must ensure a sufficiently wide surgical corridor to allow for as complete a resection as possible. To date, the surgical approach that best meets these requirements is the transcervical transparotid approach. With new technologies, including the use of robotics and endoscopy, surgery can become increasingly less invasive while maintaining the wide exposure provided by open surgical procedures.

## INTRODUCTION

The parapharyngeal space (PPS) is a fascial area located in the neck, above the hyoid bone, and below the skull base. It is bordered by several structures, including the pharynx, parotid space, uppermost nodal levels, prevertebral muscles, masticatory muscles, and submandibular space.

Parapharyngeal tumors (PPT) are rare and represent only 0.5–1% of all head and neck tumors [1,2^▪^].

PPT can originate as primary tumors or arise from the direct spread of adjacent tumors. They may also include metastatic lymph nodes, lymphoproliferative disorders, and tumors of salivary gland or neurogenic origin, among other types [3–6].

Lymph node metastases from squamous cell carcinoma (SCC) in the PPS are extremely rare lesions, accounting for about 5% of all malignant lesions diagnosed in the PPS in larger case studies [2^▪^,^7^]. In most cases described in the literature, lymph node metastases from SCC originate from the oropharynx, nasosinusal tract, and nasopharynx, with some cases reported from cutaneous SCC of the parotid region [7]. Umeda *et al.*[8] described a case series of four patients with lymph node metastases in the PPS from maxillary SCC.

The mechanism by which diseases metastasize to the PPS is not well understood, primarily because of the inaccessibility of this area, the rarity of such cases, and the limited clinical data available [9]. SCC PPS metastasis can occur in both primary and recurrence settings.

Surgical intervention remains the primary treatment for tumors in the PPS, and the intricate anatomy of this area continues to make it a challenging procedure [10]. In the case of metastatic spread to the PPS, the surgical indication should be tailored based on the individual patient and the specific type of primary or recurrent disease. Not all patients with SCC lymph node metastases in the PPS are candidates for surgery.

Various therapeutic strategies have been established to enhance both surgical and functional results. Specifically, the growing accessibility of advanced technologies has equipped surgeons with numerous options, the applications and constraints of which are still being determined [11]. 

**Box 1:**
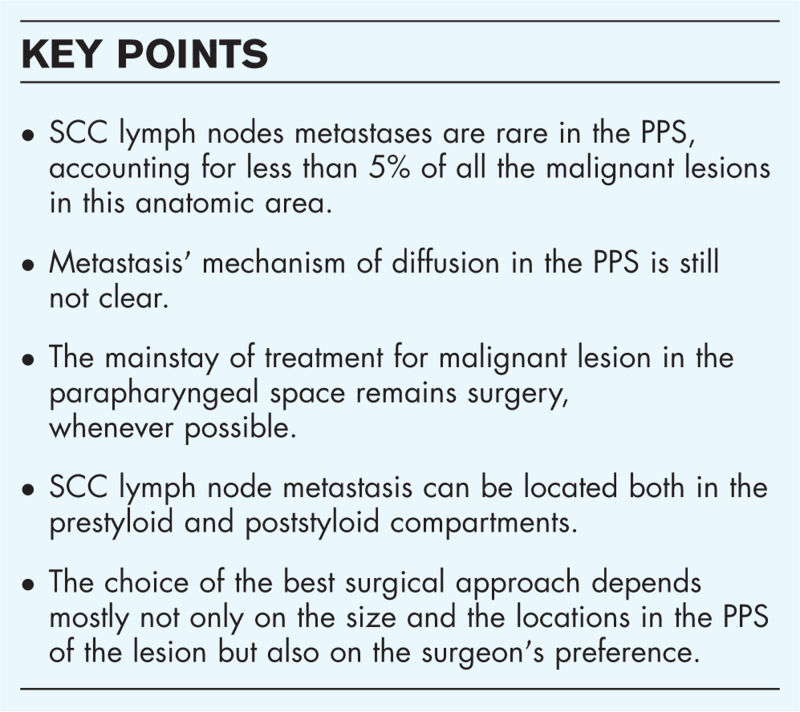
no caption available

## ANATOMY OF THE PARAPHARYNGEAL SPACE

The anatomical complexity of the PPS necessitates specific surgical approaches for managing the critical structures contained within. The various structures contained within the PPS justify the type of disease and the specific location in which it tends to manifest.

Indeed, the PPS itself can be subdivided into further subregions, each of which may be surgically targeted with specific approaches.

The PPS is shaped like an inverted triangle, extending from the skull base down to the level of the hyoid bone. The fascia that stretches backward from the styloid process to the tensor veli palatini muscle divides the PPS into anterior and posterior compartments, which are also called prestyloid and poststyloid compartments [7]. The prestyloid compartment, situated behind the pterygoid muscle, contains fatty and salivary tissues, corresponding to the deep lobe of the parotid gland. In contrast, the posterior compartment, located behind the styloid process, houses the carotid artery, internal jugular vein, cranial nerves IX, X, and XII, as well as lymph nodes [12,13,14^▪^].

This definition of the PPS is a surgical anatomical one. In fact, at the radiological level, the PPS is subdivided differently. According to a radiological division, the prestyloid space, except for the parotid gland, is defined as the true PPS, the poststyloid space is called the carotid space, and the entire parotid gland, including the deep lobe, is defined as the parotid space [15,16^▪▪^]. According to the surgical definition, the PPS encompasses the radiologically defined PPS, carotid space, and part of the parotid space, thereby encompassing a greater variety of pathologies compared to the radiological definition. The radiological definition plays a crucial role as it guides the differential diagnosis, which in turn influences clinical decision-making [16^▪▪^].

According to the classification by Prasad *et al.*[17], the PPS is divided into three compartments: the upper PPS (UPS), which extends from the skull base to the axial plane passing through the inferior border of the lateral pterygoid muscle; the middle PPS (MPS), which lies between the axial plane marking the lower limit of the UPS and a parallel plane passing through the mandibular insertion of the medial pterygoid muscle; and the lower PPS (LPS), with its inferior boundary defined by an axial plane passing through the hyoid bone. As mentioned before, the UPS and MPS were further subdivided into prestyloid compartments (or the true PPS) and retrostyloid compartments (or carotid spaces) [12]. Thus, as highlighted by Ferrari *et al.*[1], we can consider five parapharyngeal subunits: the prestyloid and retrostyloid UPS, the prestyloid and retrostyloid MPS, and the LPS.

The radiological subdivision allows for better delineation of the localization of lymph nodes within the PPS. The lymph nodes are in the carotid and parotid spaces, while they are absent in the radiologically defined PPS compartment.

Therefore, lymph node metastases can clinically only be found in the poststyloid space and in a portion of the prestyloid space [16^▪▪^]. Lymph nodes located in the poststyloid compartment of the PPS are more commonly affected by SCC of the pharynx or sinonasal region and by lymphomas [3].

## SURGICAL RESECTION OF SQUAMOUS CELL CARCINOMA METASTASIS IN THE PARAPHARYNGEAL SPACE

Based on the anatomical considerations previously discussed, lymph node metastases from SCC can be located in the poststyloid space and in a portion of the prestyloid space. The treatment varies depending on the type of primary tumor and whether it is in a primary setting or a recurrence. Therefore, in most cases, chemoradiotherapy is the first therapeutic option, considering the impact of surgery in this peculiar area. However, in recurrent tumors or those primary tumors or patients not suitable for chemoradiotherapy, surgery becomes the preferred choice. Given the anatomical complexity of the PPS and the need for a radical removal of a malignant lesion, it is crucial to choose the surgical approach that best allows access to the affected area within the PPS. Radiology plays a fundamental role in the choice of surgical approach, as it helps define the area to be treated and included in the resection. Moreover, PPS metastasis may infiltrate surrounding anatomical structures (i.e. pharynx, skull base, nerves, vessels), dictating the extent of surgical resection and the need for reconstruction or other less common procedures (i.e. internal carotid stenting or bypass).

Ferrari *et al.*[1] provided a detailed description of most of the surgical approaches available for the PPS, analyzing the main indications and limitations of each. Considering the division of the PPS into five regions proposed by Prasad *et al.*[17], the most appropriate surgical approaches have been described for each of these regions. Among the proposed algorithms for choosing the surgical approach based on disease localization in the PPS are those by Lombardi *et al.*[7] and Kanzaki *et al.*[18]. All these studies attempt to standardize the surgical choice based on imaging and the anatomical relationships between the disease and the critical structures of the PPS. However, any attempt at systematization and classification must always be considered alongside the clinical context and the individual patient's condition, and it must account for the preferences and expertise of the surgeon managing the case [1].

Lymph node metastases from SCC in the PPS can occur in its various regions. To access the upper prestyloid and poststyloid spaces, transnasal, transantral, infratemporal, and most transcervical approaches can be utilized. Lateral approaches, such as transcervical, modified transcervical–transparotid, transcervical–transparotid, transcervical–transmandibular, transparotid, transparotid–transmastoid, and infratemporal types D and C, generally offer wider working corridors with easier access and greater control over critical structures, such as vessels and nerves, particularly in the upper retrostyloid space. Favorable exposure of the MPS can be achieved with all lateral approaches. When only accessing the LPS is necessary, the transcervical approach provides the best combination in terms of exposure, working volume, tissue manipulation, and risk of bleeding.

In addition to the anatomical region where the lesion develops, it is essential to consider the lesion's histotype. For benign lesions, enucleation or excision with clear margins may suffice [19]. However, for malignant lesions, especially when lymph node metastases are present, a radical approach is mandatory, involving the removal of the entire compartment containing the disease. Therefore, to achieve wide exposure of the PPS for the removal of metastatic lymph nodes, surgical approaches that provide a wide surgical corridor and an easy maneuvering area are preferable, such as most lateral approaches to the PPS [1,17,20]. The transcervical–transparotid approach involves removing either the portion of the parotid gland below the common facial trunk or the entire parotid gland, while preserving the VII cranial nerve, to expand the surgical corridor towards the PPS [10].

Nevertheless, the transcervical–transparotid corridor remains the primary approach for adequate PPS exposure, enabling the removal of most neoplasms, including SCC lymph node metastases.^110^

Moreover, whenever the disease abuts the skull base, transcervical approaches must be combined with either transpetrous or transnasal approaches to achieve a compartmental resection aiming to oncological radicality. Thus, these extensive tumors must be managed in centers where head and neck and skull base surgeons are available to cooperate.

## POSTOPERATIVE COMPLICATIONS

The rate of postsurgical complications for PPS procedures remains quite high, ranging from 55 to 75% [6]. The main complications arising from lateral approaches to the PPS, such as the transcervical–transparotid approach, typically involve injuries to major cranial nerves or the sympathetic chains. The cranial nerves most at risk include nerves VII, IX, X, XI, and XII. In most cases, injuries to the VII cranial nerve are temporary, with nerve function typically recovering after some time [10]. Similar outcomes are observed for injuries to other cranial nerves. Malignant lesions in the PPS are associated with a higher complication rate, although there are currently no studies specifically analyzing the complications related to metastases from SCC of the PPS. Other complications include jaw pain resulting from first bite syndrome, which manifests as pain in the parotid region occurring after the first bite of a meal. Hemorrhage or stroke because of vascular injury is a serious but rare complication of PPS surgery, usually occurring when the lesion is vascular or malignant.

## OUTCOMES

Regarding the clinical and oncological outcomes of treating SCC metastases in the PPS, there are unfortunately few specific results. In fact, there are few detailed cases in the literature, and often the data are found in mixed case series where SCC nodal metastases are reported alongside primary tumors or other histologies, making it impossible to extract the specific individual data.

A recent literature review about SCC PPS metastasis has been published by Fermi *et al.*[21^▪▪^]. They analyzed the few case studies available [8,22–24] and concluded that SCC PPS metastasis can occur both in primary and recurrence settings. Through the analyzed data, the survival analysis showed that patients with recurrent SCC in the PPS had significantly worse disease-specific survival (DSS) compared with those with PPS metastasis at the time of primary tumor diagnosis and staging. However, as previously mentioned, the lack of consistent follow-up timing makes it difficult to accurately evaluate the efficacy of a management strategy. In fact, large prospective studies with specific data about patients with SCC PPS metastasis are still not available.

## FUTURE PERSPECTIVES

Future perspectives in the surgical management of the PPS involve continued exploration and refinement of innovative approaches to improve outcomes and minimize complications

Numerous studies are underway to investigate additional or combined surgical approaches to further improve access to the PPS and reduce the sequelae and potential complications related to the injury of vascular or nervous structures. Specifically, Viros *et al.*[25^▪^] demonstrated that extensive experience with transoral robotic surgery (TORS) and a deeper understanding of the endoscopic anatomy of the PPS could reduce morbidity associated with tumor resection in this dense neurovascular area.

De Virgilio *et al.*[26] carried out a systematic review to establish the current ‘state of the art’ in managing PPS tumors. The literature analysis revealed a consensus against using TORS for malignant PPS tumors, though it has been suggested for certain specific malignant cases. Currently, no study has directly compared TORS with traditional surgical methods. Consequently, the selection of a particular technique or approach is tailored to each patient, largely depending on the surgeon's preference and experience.

Meanwhile, Liu *et al.*[27^▪^] explored the potential extension of indications for using the endoscopic transoral approach compared with the traditional transoral approach. However, the indications for this approach still require thorough investigation. A key challenge for this approach is managing the internal carotid artery (ICA) in the PPS.

Zhu *et al.*[28^▪^] performed a contemporary trend analysis on the surgical management of PPS tumors at their institution and observed a growing use of endoscopy-assisted and robotic surgeries in recent years. They highlighted that endoscopy might enhance traditional approaches by offering a broader view, which facilitates the complete resection of PPS tumors and helps minimize the risks of capsule rupture or neurovascular injury.

Although these innovative approaches are being increasingly adopted, they have yet to demonstrate a clear advantage over traditional lateral accesses to the PPS, particularly when it comes to surgically treating PPS SCC metastasis.

## CONCLUSION

The treatment of PPS tumors, particularly SCC lymph node metastases, remains a controversial topic. The anatomical complexity of this region, with its numerous critical structures such as large vessels and nerves, has been extensively studied. However, surgical indication for PPS SCC lymph node metastases is still limited, typically considered in salvage surgery or when the primary tumor treatment involves surgery as the first choice. Moreover, the surgical approach for managing these cases is not yet standardized. The open transcervical transparotid approach is the most widely used, although combined approaches using endoscopy or TORS are gaining popularity. Large-scale studies focused on treating SCC lymph node metastases in the PPS are certainly needed to better analyze specific outcomes and complications.

## Acknowledgements

*None.*

### Financial support and sponsorship

*None.*

### Conflicts of interest

*There are no conflicts of interest.*

## REFERENCES AND RECOMMENDED READING

Papers of particular interest, published within the annual period of review, have been highlighted as:

▪ of special interest
▪▪ of outstanding interest

## References

[1] Ferrari M Schreiber A Mattavelli D. Surgical anatomy of the parapharyngeal space: multiperspective, quantification-based study. Head Neck 2019; 41:642–656. 30592348 10.1002/hed.25378

[2▪] Galli A Giordano L Mattioli F. The transcervical-transparotid corridor for management of parapharyngeal space neoplasms: strengths and limits in a bi-institutional retrospective series. Eur Arch Otorhinolaryngol 2024; 281:897–906. 37768370 10.1007/s00405-023-08256-7

[3] Lombardi D Nicolai P Antonelli AR. Parapharyngeal lymph node metastasis: an unusual presentation of papillary thyroid carcinoma. Head Neck 2004; 26:190–196. 14762889 10.1002/hed.10341

[4] Coskun HH Ferlito A Medina JE. Retropharyngeal lymph node metastases in head and neck malignancies. Head Neck 2011; 33:1520–1529. 20737485 10.1002/hed.21526

[5] Bozza F Vigili MG Ruscito P. Surgical management of parapharyngeal space tumours: results of 10-year follow-up. Acta Otorhinolaryngol Ital 2009; 29:10–15. 19609376 PMC2689563

[6] Riffat F Dwivedi RC Palme C. A systematic review of 1143 parapharyngeal space tumors reported over 20 years. Oral Oncol 2014; 50:421–430. 24589290 10.1016/j.oraloncology.2014.02.007

[7] Lombardi D Ferrari M Paderno A. Selection of the surgical approach for lesions with parapharyngeal space involvement: a single-center experience on 153 cases. Oral Oncol 2020; 109:104872. 32659725 10.1016/j.oraloncology.2020.104872

[8] Umeda M Minamikawa T Yokoo S Komori T. Metastasis of maxillary carcinoma to the parapharyngeal space: rationale and technique for concomitant en bloc parapharyngeal dissection. J Oral Maxillofac Surg 2002; 60:408–413. 11928098 10.1053/joms.2002.31253

[9] Sandler ML Xing MH Levy JC. Metastatic thyroid carcinoma to the parapharyngeal and retropharyngeal spaces: systematic review with seven newly reported cases describing an uncommon presentation of a common disease. Head Neck 2021; 43:1331–1344. 33295689 10.1002/hed.26572

[10] Fermi M Serafini E Ferri G. Management of parapharyngeal space tumors with transparotid–transcervical approach: analysis of prognostic factors related with disease-control and functional outcomes. Eur Arch Otorhinolaryngol 2022; 279:2631–2639. 34529157 10.1007/s00405-021-07074-z

[11] Paderno A Piazza C Nicolai P. Recent advances in surgical management of parapharyngeal space tumors. Curr Opin Otolaryngol Head Neck Surg 2015; 23:83–90. 25692627 10.1097/MOO.0000000000000134

[12] Stambuk HE Patel SG. Imaging of the parapharyngeal space. Otolaryngol Clin North Am 2008; 41:77–101. 18261527 10.1016/j.otc.2007.10.012

[13] Strohl MP El-Sayed IH. Contemporary management of parapharyngeal tumors. Curr Oncol Rep 2019; 21:103. 31728649 10.1007/s11912-019-0853-8

[14▪] Jiang C Wang W Chen S Liu Y. Management of parapharyngeal space tumors: clinical experience with a large sample and review of the literature. Curr Oncol 2023; 30:1020–1031. 36661727 10.3390/curroncol30010078 PMC9857702

[15] Harnsberger HR Osborn AG. Differential diagnosis of head and neck lesions based on their space of origin. 1. The suprahyoid part of the neck. Am J Roentgenol 1991; 157:147–154. 2048510 10.2214/ajr.157.1.2048510

[16▪▪] Rigsby RK Bhatt AA. Primary pathology of the parapharyngeal space. Clin Neuroradiol 2023; 33:897–906. 37380900 10.1007/s00062-023-01316-9

[17] Prasad SC Piccirillo E Chovanec M. Lateral skull base approaches in the management of benign parapharyngeal space tumors. Auris Nasus Larynx 2015; 42:189–198. 25270862 10.1016/j.anl.2014.09.002

[18] Kanzaki S Nameki H. Standardised method of selecting surgical approaches to benign parapharyngeal space tumours, based on preoperative images. J Laryngol Otol 2008; 122:628–634. 17655777 10.1017/S0022215107009875

[19] Nicolai P Paderno A Farina D Piazza C. Microdebrider cavitation and transcervical removal of parapharyngeal schwannomas approaching the skull base. Eur Arch Otorhinolaryngol 2014; 271:3305–3311. 24584553 10.1007/s00405-014-2953-2

[20] Marzouki H Nujoom M Fagih SN. Surgical parapharyngeal space tumor analysis with case series study. Comput Intell Neurosci 2022; 2022:7083240. 35198022 10.1155/2022/7083240 PMC8860510

[21▪▪] Fermi M Botti C Chiari F. Squamous cell carcinoma metastatic to the lymph nodes of the parapharyngeal space: case series and systematic review. Acta Otorhinolaryngol Ital 2024; 44:223–232. 39347547 10.14639/0392-100X-N2993 PMC11441514

[22] Dallan I Fiacchini G Turri-Zanoni M. Endoscopic-assisted transoral-transpharyngeal approach to parapharyngeal space and infratemporal fossa: focus on feasibility and lessons learned. Eur Arch Otorhinolaryngol 2016; 273:3965–3972. 27139702 10.1007/s00405-016-4074-6

[23] Dimitrijevic MV Jesic SD Mikic AA. Parapharyngeal space tumors: 61 case reviews. Int J Oral Maxillofac Surg 2010; 39:983–989. 20638245 10.1016/j.ijom.2010.06.005

[24] Douglas WG Rigual NR Giese W. Advanced soft palate cancer: the clinical importance of the parapharyngeal space. Otolaryngol Head Neck Surg 2005; 133:66–69. 16025055 10.1016/j.otohns.2005.03.007

[25▪] Virós Porcuna D Pollán Guisasola CM Viña Soria C. Transoral robotic parapharyngeal space dissection. Head Neck 2024; 46:2657–2660. 39132821 10.1002/hed.27902

[26] De Virgilio A Costantino A Mercante G. Trans-oral robotic surgery in the management of parapharyngeal space tumors: a systematic review. Oral Oncol 2020; 103:104581. 32058293 10.1016/j.oraloncology.2020.104581

[27▪] Liu J Liu Q Sun XC. Endoscopic transoral approach to the parapharyngeal space: technical nuances and preliminary results. J Laryngol Otol 2023; 137:678–684. 35791870 10.1017/S0022215122001621

[28▪] Zhu X Shi X Zhou L. Trends in the surgical management of parapharyngeal space tumors: a single-center retrospective analysis. Eur J Surg Oncol 2023; 49:47–54. 36089451 10.1016/j.ejso.2022.08.016

